# Decoding altitude-activated regulatory mechanisms occurring during apple peel ripening

**DOI:** 10.1038/s41438-020-00340-x

**Published:** 2020-08-01

**Authors:** Evangelos Karagiannis, Michail Michailidis, Georgia Tanou, Federico Scossa, Eirini Sarrou, George Stamatakis, Martina Samiotaki, Stefan Martens, Alisdair R. Fernie, Athanassios Molassiotis

**Affiliations:** 1 Laboratory of Pomology, Department of Agriculture, Aristotle University of Thessaloniki, 54124 Thessaloniki, Greece; 2 Institute of Soil and Water Resources, ELGO-DEMETER, Thermi, Thessaloniki, 57001 Greece; 3 Max-Planck-Institute of Molecular Plant Physiology, Am Müehlenberg 1., Potsdam-Golm, 14476 Germany; 4 Council for Agricultural Research and Economics, Research Center for Genomics and Bioinformatics, Via Ardeatina 546, 00178 Rome, Italy; 5 Institute of Plant Breeding and Genetic Resources, ELGO-DEMETER, Thermi, Thessaloniki, 57001 Greece; 6 Biomedical Sciences Research Center “Alexander Fleming”, Vari, 16672 Greece; 7 Fondazione Edmund Mach, Centro Ricerca e Innovazione, Department of Food Quality and Nutrition, Via E. Mach, 1, 38010 San Michele all’Adige, TN Italy

**Keywords:** Proteomics, Metabolomics

## Abstract

Apple (*Malus domestica* Borkh) is an important fruit crop cultivated in a broad range of environmental conditions. Apple fruit ripening is a physiological process, whose molecular regulatory network response to different environments is still not sufficiently investigated and this is particularly true of the peel tissue. In this study, the influence of environmental conditions associated with low (20 m) and high (750 m) altitude on peel tissue ripening was assessed by physiological measurements combined with metabolomic and proteomic analyses during apple fruit development and ripening. Although apple fruit ripening was itself not affected by the different environmental conditions, several key color parameters, such as redness and color index, were notably induced by high altitude. Consistent with this observation, increased levels of anthocyanin and other phenolic compounds, including cyanidin-*3-O*-galactoside, quercetin-*3-O*-rhamnoside, quercetin-*3-O*-rutinoside, and chlorogenic acid were identified in the peel of apple grown at high altitude. Moreover, the high-altitude environment was characterized by elevated abundance of various carbohydrates (e.g., arabinose, xylose, and sucrose) but decreased levels of glutamic acid and several related proteins, such as glycine hydroxymethyltransferase and glutamate–glyoxylate aminotransferase. Other processes affected by high altitude were the TCA cycle, the synthesis of oxidative/defense enzymes, and the accumulation of photosynthetic proteins. From the obtained data we were able to construct a metabolite-protein network depicting the impact of altitude on peel ripening. The combined analyses presented here provide new insights into physiological processes linking apple peel ripening with the prevailing environmental conditions.

## Introduction

Apple (*Malus* x *domestica* Borkh), with its wide diversity of climatic adaptation, has become the most widely planted tree fruit of the temperate zone and one of the most widely cultivated in the world^1^. The ability of apple genotypes to produce different phenotypes as a function of environmental cues (so-called phenotypic plasticity) is considered one of the main processes by which this fruit species can face and adapt to the spatio-temporal variation of environmental factors^1^. In addition, apple is being increasingly considered as a model species for fruit development studies. These facts aside, many gaps still exist in apple research^2^.

Three distinct parts can be distinguished in apple fruit: the outer pericarp (peel), the mesocarp (flesh), and the core. The outer pericarp and the mesocarp constitute the pericarp that develops and ripens, reflecting a deep reprogramming in the metabolic shift^3,4^. Many apple fruit quality characteristics appear to be specifically related to only one of the two pericarp tissues. For instance, taste-related traits such as the sugar and acid content mostly depend on the flesh^5^, while the apple peel is the main site in the synthesis of many compounds of interest (e.g. anthocyanins and aroma volatiles), indicating that these tissues undergo specific ripening processes^6^. Thus, the characterization of peel tissue, regarding its ability to sense environmental stimuli, would appear to be an essential route by which to enhance our understanding of apple fruit ripening^7^.

Apple fruits growing along an altitudinal gradient are excellent resources to study the influence of environment on various developmental and ripening processes. This is because several environmental factors are altered at different altitudes, such as night/day temperature, UV radiation, and light intensity, or even a combination of all these parameters, could alter the processes of apple development^8^. For example, several studies have firmly established that temperature and light are the most crucial environmental factors affecting the manifestation and development of red color in apples^9^. Similarly, heating apple fruit rapidly reduced the expression of the R2R3 MYB transcription factor (*MYB10*), which is responsible for the coordinative regulation of the red peel color, as well as for the expression of other genes in the transcriptional activation complex^10^. Bai et al. (2014) also demonstrated that UV‐B induces the expression of the B‐box (BBX) gene *MdCOL11* in apple and that the *MdCOL11* promoter is targeted by MdHY5 to promote anthocyanin synthesis in apple peel^11^ It is widely accepted that the majority of these environmental factors (i.e., temperature and light) are tightly associated with altitude, therefore altitude could be an important parameter involved in the regulation of the final ripening metabolism of apple peel tissue^11,12^.

High-throughput molecular biological techniques, such as transcriptomic, proteomic, and metabolomic approaches, have been widely used to explore ripening-related mechanisms in fruits, including apples^4,11,13,14^. Moreover multi-omics analyses of apple peel have recently been conducted in our research group^15,16^. However, multi-omics studies of peel ripening in association with altitude changes have not been conducted yet. In the present study, using integrated metabolomic and proteomic analysis, we aimed to obtain a comprehensive understanding of the ripening expression pattern of peel tissue in Fuji apple (‘Fujiku’) growing at two different altitudes, designated as low (20 m) and high (750 m) altitude.

The Fuji apple represents a suitable model material, since this widespread cultivar is cultivated in various altitudes and has a great economic and nutritional value. Together with fruit phenotypic plasticity, particularly regarding appearance traits, various photosynthetic and oxidative stress proteins, carbohydrates, components of glutamic-acid metabolism, anthocyanin, and other phenolic compounds were found to be differentially abundant between the low and high altitude. Based on these results, we propose a model of a protein-metabolic network underlying the dynamics of apple peel ripening in response to distinct altitudes.

## Results

### Phenotypical and physiological traits of apple fruit grown in distinct environmental conditions

**Fig. 1:**
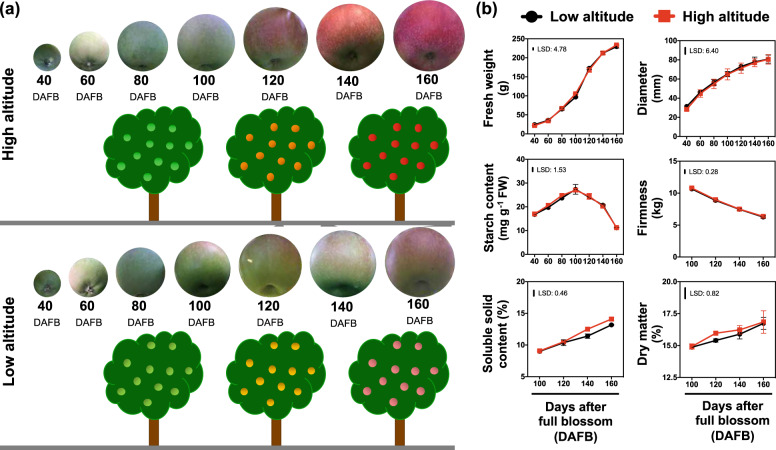
Sampling strategy and the effect of altitude on apple ‘Fuji’ fruit ripening. **a** Fruits were collected at different developmental stages (40, 60, 80, 100, 120, 140, and 160 days after full blossom; DAFB) from two orchards located in low (20 m) and high (750 m) altitude region. **b** The impact of altitude on apple ripening features, namely fresh weight (g), fruit diameter (mm), starch content (mg g^−1^ FW), firmness (kg), soluble solid content (%), and dry matter (%). The vertical bar represents the least significant difference (LSD) at 5% level of significance, of three independent biological measurements, which was used for means comparison between the different altitudes (low- and high-) and timepoints (DAFB), while the vertical bars at each timepoint represent the standard error of the means (SEM)

**Fig. 2:**
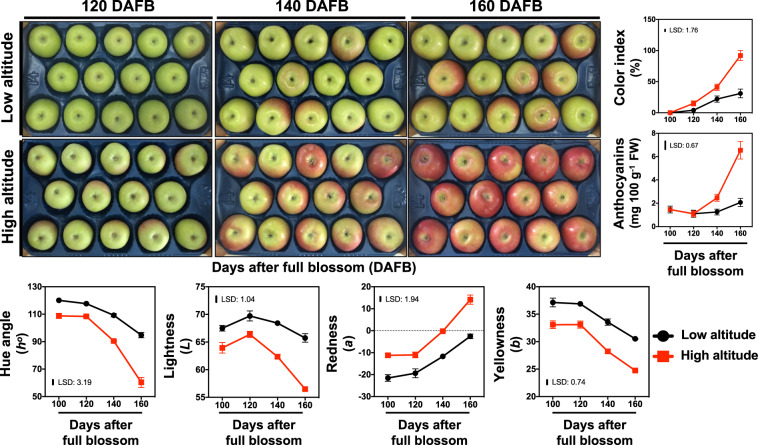
Phenotypic plasticity and color changes in apples fruit exposed to different altitude conditions. The impact of altitude on the phenotype at three final ripening stages (120, 140, and 160 days after full blossom; DAFB), color index (%), anthocyanins content (mg 100 g^−1^ FW), hue angle (*h*^*o*^), lightness (*L*), redness (*a*) and yellowness (*b*) between the two environmental distinct regions (20 and 750 m). The vertical bar represents the least significant difference (LSD) at 5% level of significance, of three independent biological measurements, which was used for means comparison between the different altitudes (low- and high-) and timepoints (DAFB), while the vertical bars at each timepoint represent the standard error of the means (SEM). Specifically, metabolites of increased abundance at high altitude were the carbohydrate sugars arabinose, xylose, sucrose, turanose, talose, as well as citric acid (organic acid) and lactitol (polyol) (Fig. 3, Supplementary Table 1). Meanwhile, metabolites with decreased abundance at high altitude were the organic acids fumarate, quinate, and malate, the carbohydrate sugars rhamnose and fucose, as well as the amino acid glutamic acid (Fig. 3, Supplementary Table 1)

The phenotype of apple fruit across development (Fig. 1a; the same fruits were photographed across seven different timepoints throughout development), and especially during the commercial harvest stage (Fig. 2) indicates that altitude and its associated climatic factors have a strong impact on apple peel color. In order to characterize the relationship between altitude and apple fruit development we analyzed various parameters from both cultivation regions. Data showed that apple fruits sampled at various developmental stages from the two environmental conditions displayed a similar pattern of growth (fruit diameter fresh weight, dry matter) and ripening (starch content, firmness, soluble solids content) (Fig. 1b)—with these data suggesting, that the ripening status of the fruits was unaffected by altitude. However, apple peel color, assessed by various parameters including *L*, *a*, *b*, and *Hue angle*, was remarkably altered by the different environmental conditions (Fig. 2). In keeping with this, the percentage (%) of outer pericarp color was more than doubled in the high-altitude environment at 120, 140, and 160 days after full bloom (DAFB), reaching up to 92% of the apples surface at harvest stage (160 DAFB). In addition, the anthocyanin content followed the same pattern observed for color index between the two regions (Fig. 2).

### Metabolic profiling in apple peel during various developmental stages at different altitudes

**Fig. 3:**
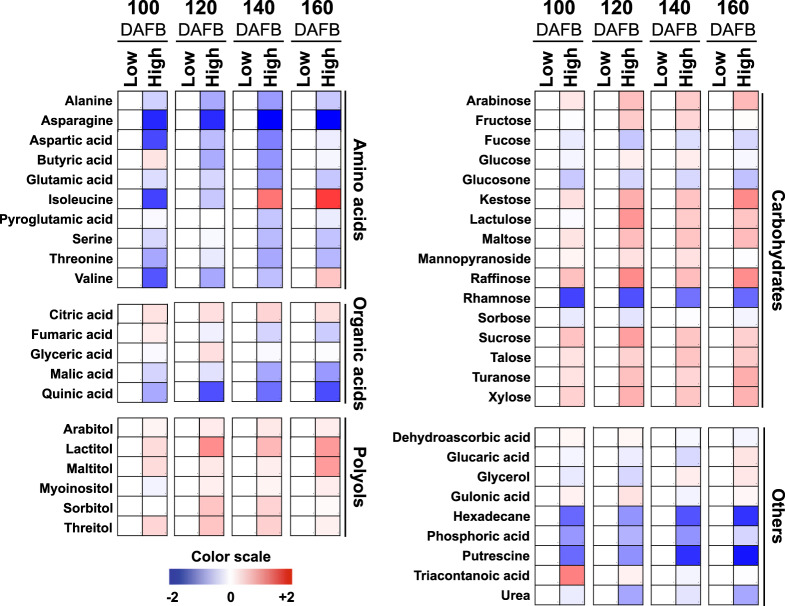
Modulation of apple peel primary metabolites across development between low- and high-altitude environmental regions. Changes in primary metabolites in peel tissue at 100, 120, 140, and 160 days after full blossom (DAFB) between low- and high-altitude regions (20 m and 750 m). A color scale that is proportional to the log2 ratio of each identified metabolite shows the fold-change between low- and high-altitude regions. Mean values of three independent measurements for each region were analyzed between the distinct environments. Relative values for each metabolite mean are provided in Supplementary Table 1

**Fig. 4:**
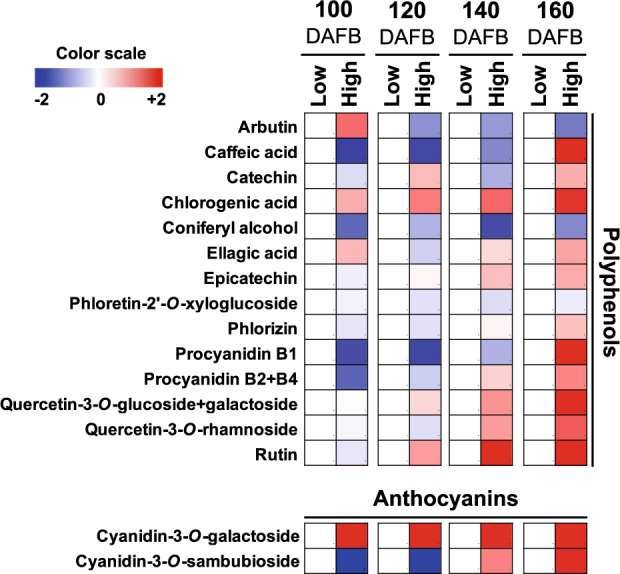
The impact of low- and high-altitude in the levels of peel secondary metabolites across apple fruit development. Changes in secondary metabolites in peel tissue at 100, 120, 140, and 160 days after full blossom (DAFB) between low- and high- altitude regions (20 m and 750 m). A color scale that is proportional to the log2 ratio of each identified metabolite shows the fold-change between low- and high-altitude regions. Mean values of three independent measurements for each region were analyzed between the distinct environments. Relative values for each metabolite mean are provided in Supplementary Table 2

To obtain information concerning the effect of altitude on peel metabolism, analysis of both primary and secondary metabolites was performed in low- and high-altitude environment cultivated apples at 140 (preharvest) and 160 (harvest) DAFB, (Figs. 3, 4, Supplementary Table 1 and 2). We observed that the accumulation level of thirteen primary metabolites varied at preharvest and harvest periods between the two environments, with seven and six metabolites showing increased and decreased abundance at high-altitude region, respectively (Fig. 3).

Analysis of secondary metabolites further indicated that among the 16 polyphenolic metabolites detected in both environments, six were significantly altered between the two regions during preharvest and harvest periods (Fig. 4). In detail, the steady-state level of chlorogenic acid, quercetin-3-*O*-rutinoside (rutin), epicatechin, quercetin-3-*O*-rhamnoside (quercetin-3-rha), quercetin-3-*O*-glucoside+galactoside (SUM) and the anthocyanins cyanidin-3-*O*-galactoside (Cy-3-gal), and cyanidin-3*-O-*sambubioside were increased in the peel tissue of fruit grown at high altitude (Fig. 4, Supplementary Table 2).

### Identification and quantification of apple peel proteins

To characterize the different apple peel phenotypes (Figs. 1a, 2), a comparative proteomic analysis of the peel tissue was conducted at 140 and 160 DAFB stages. A total number of 3370 proteins were confidently identified. The identified proteins were classified into 13 functional categories and 14 subcellular localizations (Supplementary Fig. 2, Supplementary Table 3).

**Fig. 5:**
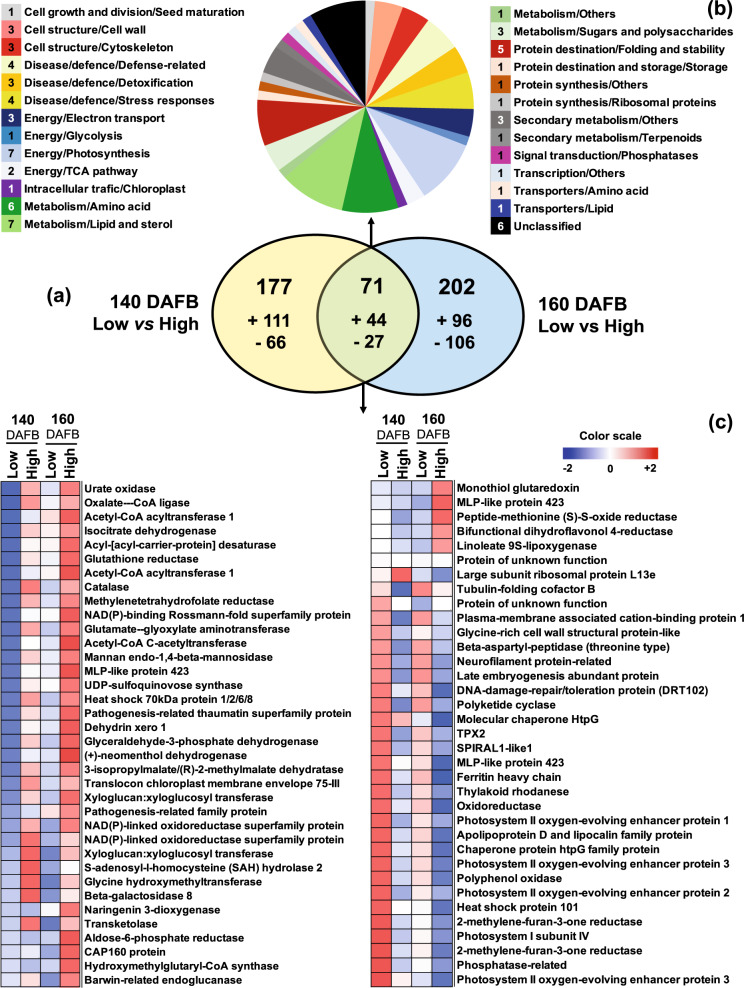
Temporal accumulation pattern of altitude-affected apple peel proteins at different ripening stages. **a** Functional categorization of altitude-affected proteins (71 proteins; see below in Fig. 5b) characterized as overlapping between preharvest (140 DAFB) and harvest (160 DAFB) timepoints. The color code represents the functional classes of the identified proteins. The total number in each set of proteins is shown. **b** Venn diagrams indicating the number of altitude-affected proteins at preharvest (177 proteins) and harvest (202 proteins) time, and the overlap between these conditions (71 proteins). The ‘plus’ or ‘minus’ symbols indicate the number of upregulated or downregulated proteins, respectively. **c** Heat diagram showing the accumulation profiles of overlapping proteins presented in Fig. 5b. A color scale that is proportional to the log2 ratio of each identified protein shows the fold-change between low- and high-altitude regions. Mean values of three independent biological and three technical measurements for each region were analyzed between the distinct environments. Additional details for each protein are given in Supplemental Tables 3 and 4

To examine specific protein changes in response to the different altitude environments we focused on proteins that were commonly affected between low- and high-altitude regions at both developmental stages (140 and 160 DAFB). Αt 140 DAFB stage, there were 34 proteins whose abundance was increased in the high-altitude region, while 37 proteins were decreased, respectively (Fig. 5c, Supplementary Table 4). Moreover, at 160 DAFB there were 28 proteins, which were downregulated and 43 proteins, which were upregulated in the high-altitude environment (Fig. 5c, Supplementary Table 4). In addition, high altitude resulted in the suppression of 27 common proteins at both stages, while 33 proteins were increased at both developmental timepoints (Fig. 5c, Supplementary Table 4). Furthermore, the high-altitude region suppresses the abundance of 10 proteins at preharvest stage, while the same proteins were upregulated at harvest stage. Finally, one protein (large subunit ribosomal protein L13e) sub-localized to the chloroplast was upregulated in the high-altitude region during preharvest stage but was subsequently downregulated at harvest time (Fig. 5c, Supplementary Table 4).

## Discussion

Apple fruit undergoes a broad metabolic adaption and unreversed specialization during the fruit ripening progress, which is known to involve internal signals refined by environmental cues^13^. Essentially all of what is known about apple fruit development pertains to the changes of mesocarp or of the whole fruit, and little is known about the function of the peel tissue, as an isolated component, during apple ripening. In addition to this, our current knowledge about the influence of altitude on apple peel is entirely derived from research on fruit coloration-related aspects of ripening^3,4,6,11^. Therefore, an overall molecular framework is needed for better understanding of the altitude-associated changes in metabolism during peel ripening.

In this study, we characterized the impact of discrete environments (altitudes) on apple peel metabolism. It is notable that apples sampled at the two different altitudes exhibited a similar pattern of key ripening traits, including fresh weight, starch content, fruit diameter, firmness, soluble solids content, and dry matter (Fig. 1b). This evidenced that the sampled fruit at both environmental conditions displayed the same ripening status; therefore, the observed phenotypes (Figs. 1a, 2) cannot be due to an advance/delay in fruit ripening time but rather has to be related to differences in altitude-driven regulation of ripening. It is, however, worth noting that the different growth conditions do influence several peel color parameters, namely the hue angle (*h*^*o*^), lightness (*L*), redness (*a*) yellowness (*b*) as well as the total anthocyanin level and the percentage (%) of peel color index (Fig. 2), verifying previous findings that the red coloration in apple and other fruit is stimulated by altitude^6,7^. These differences between apples grown at the two distinct altitude cultivation regions could be a result of specific metabolic and biochemical adjustments to the environmental factors, such as day length, ambient light, temperature, or even an interplay between these parameters collectively affecting apple peel biology. For example, the sensitivity and responsiveness of anthocyanin biosynthesis to temperature was demonstrated in an on-tree experiment with ‘Mondial Gala’ and ‘Royal Gala’ apples where a single night of lower temperature was sufficient to elicit a large increase in transcription of *MYB10* and consequently also in anthocyanin abundance^10^.

**Fig. 6:**
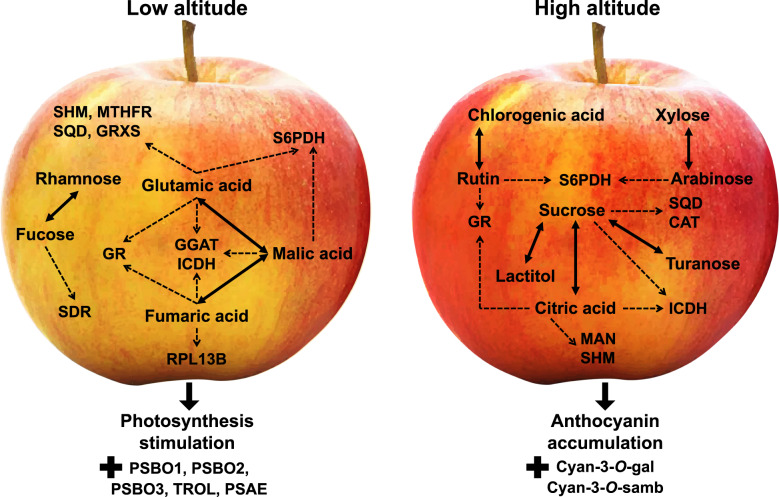
A developmental model of key metabolite-protein changes in apple peel associated with the altitudinal gradient. Schematic representation of a metabolite-protein network that particularly induced in each altitude. The ‘—’ and ‘---’ arrows indicate the metabolite and protein connection to the network, respectively. ICDH isocitrate dehydrogenase, GR glutathione reductase, CAT catalase, MTHFR methylenetetrahydrofolate reductase, GGAT glutamate–glyoxylate aminotransferase, MAN, mannan endo-1,4-beta-mannosidase, SQD UDP–sulfoquinovose synthase, SDR (+)-neomenthol dehydrogenase, SHM glycine hydroxymethyltransferase, S6PDH aldose-6-phosphate reductase, GRXS monothiol glutaredoxin, RPL13B large subunit ribosomal protein L13e, PSBO1 photosystem II oxygen-evolving enhancer protein 1, PSBO2 photosystem II oxygen-evolving enhancer protein 2, PSBO3 photosystem II oxygen-evolving enhancer protein 3, TROL thylakoid rhodanese, PSAE photosystem I subunit IV, Cy-3*-O-*gal cyanidin-3*-O-*galactoside, Cyan-3*-O-*samb cyanidin-3*-O-*sambubioside

It was previously shown that numerous environmental signals induced major metabolic changes during fruit ripening^17^. Data from our current study indicates that apples grown at high altitude provoke the accumulation of specific carbohydrates, such as arabinose, xylose, sucrose, turanose, and talose at distinct developmental stages (Fig. 3). Our results, when taken together with previous ones^18^, suggest a crucial roles of carbohydrate biosynthesis in response to high altitude. In this regard, an interesting observation was the greater accumulation of SQD (UDP–sulfoquinovose synthase), that is related to sucrose accumulation^19^, in apple fruit grown at high altitude (Figs. 3, 6). This observation suggests that cell-wall loosening across apple peel development would provide arabinose and xylose, as the major neutral sugars that contribute carbon to the enlarging cells towards environmental stimuli^14,20^. This observation is supported by present data showing that xyloglucan–xyloglucosyl transferase (XTH), a protein which catalyzes fundamental cell-wall properties^21^, and is subsequently involved in the biosynthesis of xylose/arabinose in peel cells was induced by high altitude.

Another interesting observation made in our study was the decrease in fucose and especially rhamnose at high altitude, with the simultaneous accumulation of (+)-neomenthol dehydrogenase (SDR), an enzyme active in reduction-oxidation- processes of the plant cell^22^ (Figs. 3, 6; Supplementary Table 1). The reduction of a rhamnose biosynthetic enzyme at high altitude indicates that the apple peel exhibits a loss in its capacity to convert the newly synthesized polyuronides to a more tightly bound form compared to low-altitude environments. The accumulation of these compounds at high altitude might play a key role in the enrichment of the outer cell membrane due to the higher temperatures during fruit development, as already proposed in peach peel^7^. However, further experiments are required in order to substantiate this hypothesis.

The present study also showed that both the citrate level and isocitrate dehydrogenase (ICDH) abundance were increased in fruit exposed to high altitude (Figs. 3, 5c; Supplementary Table 4). Given that the citrate and isocitrate conversions play important roles in TCA metabolism^23^, these findings might be suggestive of a general increase of TCA cycle activity, and, consequently, a likely induction of the mitochondrial levels of NADH and NADPH leading to alterations in apple fruit pigment content (Fig. 2). Evidence for such a compensatory mechanism has been provided by expressing a fragment of the mitochondrial NAD-dependent isocitrate dehydrogenase gene (*Sl*IDH1) in tomato^24^. The transgenic lines exhibited considerably altered metabolism, being characterized by altered TCA flux, NAD(P)H levels, and leaf pigmentation^24^.

In addition to primary metabolism, we also focused on secondary metabolites that are known to be important for apple fruit biology and quality^25^. Data revealed a widespread accumulation of various polyphenolic compounds, including quercetin-3-*O*-rhamnoside, quercetin-3-*O*-glucoside+galactoside (SUM), quercetin-3-*O*-rutinoside, and chlorogenic acid in apple peel at high-altitude environment (Fig. 4, Supplementary Table 2). In addition, chlorogenic acid and epicatechin, as major phenolic compounds in several apple cultivars, including ‘Fuji’^26,27^, were also stimulated by high altitude (Fig. 4, Supplementary Table 2), indicating an environment-based regulation of these phenolic metabolites. Previous studies in apple peel demonstrated that the accumulation of anthocyanin cyanidin-3-*O*-galactoside was induced by low temperatures, and thus promoted fruit coloration by up-regulating the expression of genes involved in anthocyanin biosynthesis^28^. In the present study, the anthocyanin Cy-3-gal and cyanidin-3-*O*-sambubioside were increased in apple grown at high altitude (Fig. 4, Supplementary Table 2). It is interesting that cyanidin-3-*O*-sambubioside is one of the major anthocyanin compounds in black peanut skin^29^; however, till now, no information is available to the impact of cyanidin-3-*O*-sambubioside on apple peel metabolism. The greater accumulation of this anthocyanin at high-altitude grown apples, might therefore, be related to the higher abundance of the total anthocyanin levels in apple peel (Fig. 2). Apple fruits, and especially peel tissues, at high altitude can counteract severe environmental conditions such as high UV radiation, low temperature, large variations in the day–night temperature, etc. as compared to the low-altitude grown fruit. Thus, fruit growing at high-altitude environment may develop strategies in order to adapt to these conditions. For example, UV light has a sustained effect on anthocyanin and flavonol production via regulation of HY5 and MYB10/MYB22 in ‘Royal Gala’ apple fruit^30^.

Another major target of interest in this study was the changes of peel protein profiles in response to altitude. In particular, the high-altitude-dependent induction of linoleate 9S-lipoxygenase reported here (Fig. 5c, Supplementary Table 4) is indicative of the involvement of this protein in regulating the synthesis of flavor compounds. Consistent with this, the strong accumulation of isoleucine at high altitude (Fig. 3) could provide precursors to branched-chain esters and may contribute to the formation of short-chain fatty acids^31^, which are linked to the lipoxygenase pathway and thereby to apple aroma composition. As such these results seem to imply a diverse role of the lipoxygenase pathway in apple fruit across different environmental conditions.

Apple fruit undergo a physiological transition on the differentiation of photosynthetically active chloroplasts into chromoplasts during ripening, and this transition would appear to be coupled to a decline in enzymatic activities associated with carbon assimilation^32^. In this work, the abundances of several photosynthetic proteins, including photosystem II oxygen-evolving enhancer protein 1, photosystem II oxygen-evolving enhancer protein 2, photosystem II oxygen-evolving enhancer protein 3, thylakoid rhodanese, and photosystem I subunit IV were repressed by high altitude (Fig. 5c). However, this photosynthetic-related repression had remarkably little effect on either fruit growth dynamics or sugar accumulation (Fig. 1a), which suggests that the vast majority of photoassimilates are supplied by the leaves rather than produced de novo in the apple fruit. This is in-line with the results of a previous study^33^, where it was shown that transgenic tomato plants exhibiting decreased expression of glutamate 1-semialdehyde aminotransferase were characterized by a reduced photosynthetic activity, as determined by the levels of intermediates of the Calvin–Benson cycle, but without effects on fruit growth and ripening. The authors suggested that under certain environmental conditions (i.e. higher light irradiance), the repression of fruit photosynthetic capacity could probably be compensated by an enhanced import of photoassimilates from source tissues^33^. The observed reduction of these photosynthetic proteins (Fig. 5c) is coincident with the loss of green color during chlorophyll degradation and the red coloration in apple fruit during maturity transition (Figs. 1a, 2). Indeed, both are potentially linked to the increased anthocyanin content of the high-altitude fruit (Figs. 2, 4), suggesting the presence of tight regulatory control between photosynthetic proteins and anthocyanin biosynthesis. The link between photosynthesis/anthocyanin homeostasis and fruit development is by no means unprecedented. Previous data proposed that anthocyanins protect photosynthetic tissues by shading chloroplasts from blue–green light^34^, while anthocyanins have been shown to reduce photobleaching and to decrease photoinhibition in the apple peel exposed low temperature^35^.

Oxidative stress in the fruit is known to increase markedly during the last stages of normal fruit development; and this has been proposed to be part of the ripening process with the aim of facilitating metabolic changes associated with this phenomenon^17^. During fruit ripening, chloroplasts develop into chromoplasts, accumulating high amounts of antioxidant compounds, which presumably will protect the chromoplast and fruit cells^32^. In this sense, the massive peel coloration at high altitude (Figs. 1a, 2) was accompanied with a strong increase in the abundance of several oxidative stress-related proteins, including catalase, ascorbate peroxidase, glutathione reductase, and monodehydroascorbate reductase as well as of various defense-related proteins such as the heat shock 70 kDa protein 1/2/6/8 and pathogenesis-related thaumatin superfamily proteins (Fig. 5c, Supplementary Table 4). Thus, it is possible that the high-altitude environment may alter defense metabolism, thereby enabling apple peel to withstand these conditions.

An important result revealed by our study was that the high-altitude peel tissues had substantially lower fumarate, malate and glutamic-acid contents than those grown at low altitude (Figs. 3, 6). Using protein-metabolite correlation network analysis (Supplementary Fig. 3), we also found that the reduction of these metabolites was accompanied with the decrease of several glutamic-acid-related proteins such as glycine hydroxymethyltransferase (SHM), methylenetetrahydrofolate reductase (MTHFR), monothiol glutaredoxin (GRXS), glutamate–glyoxylate aminotransferase (GGAT), aldose-6-phosphate reductase (S6PDH), and the large subunit ribosomal protein L13e (RPL13B) (Figs. 5c, 6; Supplementary Table 4). Although very little is known about glutamate fluxes and the dynamics of apple peel tissues, it seems possible that glutamate could participate in intercellular signaling^36^. There is preliminary evidence that environmental factors trigger glutamate efflux into the apoplast to activate glutamate-gated channels, causing a transient change in [Ca^2+^]_cyt_ and a cascade of downstream responses in *Arabidopsis*^37^. Consistent with this possibility, plant tissues have evolved the capacity to respond to variations in glutamate concentration in the external environment^38^. Moreover, the observation that isocitrate dehydrogenase (ICDH) is present at higher levels in high-altitude fruit (Fig. 5 and Fig. 6) along with the established role of this enzyme in glutamate biosynthesis^36^, prompts the question as to whether this metabolic route has an undescribed function in apple peel tissues that is associated with the altitudinal gradient. However, more work is needed to characterize the biological function of the proposed network (Fig. 6). For example, functional genomics analysis alongside the application of artificial methods to change specific climatic characteristic (e.g., light, temperature), would likely allow one to verify how altitude environmental factors may influence the whole-peel metabolism during apple ripening transition.

## Conclusion

This work provides insights into the metabolic shifts occurring in apple peel across development in distinct growth environments. Data revealed that low altitude is able to produce an apple fruit with equal quality traits to those grown at high-altitude regions, with the exception of a strong red peel color. This implies that apple flesh and peel function in a ripening-autonomous manner. We demonstrate here that each altitude was related to specific changes in proteins and metabolites across fruit development. On this basis, we propose a model that explains how low-altitude is linked with several key ripening events, including the abundance of various photosynthetic proteins (e.g., PSBO1/2/3, TROL, and PSAE) and the status of glutamic-acid-related metabolism. On the other hand, high altitude induced both anthocyanin (Cy-3-*O*-gal and Cyan-3-*O*-samb) and carbohydrate (e.g., sucrose, xylose, and arabinose) biosynthesis during apple peel ripening (Fig. 6; see text for details). It is interesting to note that orchard altitude has a profound impact on temperature, light, humidity, and other factors, which have a direct influence on apple peel metabolism. Based on the previous published work in apple fruit^5,6,11,28^ as well as on the meteorological data from the two sites studied here (Supplementary Fig. 1) we can assume that most of the color-related phenotypic variability can be associated with the different air temperature and UV levels, both of which are strongly related to altitude^3,4,7^. Since the overall peel metabolism is controlled by several parameters, it is, however, unlikely that the entire altitude-associated metabolic changes (Fig. 6) can be related to a single environmental factor. Extensive further work in order to identify the specific environmental stimuli involved in apple peel ripening metabolism is therefore required to provide further insight into the mechanisms underlying our observations.

## Material and methods

### Fruit material and peel tissue sampling

In order to investigate the effect of altitude on apple fruit physiology, outer pericarp (peel) tissue samples of cv. ‘Fujiku’ were collected from two independent experimental orchards in two regions with significant difference in altitude. The first orchard was located at Alexandreia (Imathia, North Greece) at about 20 m altitude (defined as low altitude), and the second orchard located at Pyrgoi (Kozani, North Greece) at about 750 m altitude (defined as high altitude). The mean monthly air temperature of both orchards is provided as supplementary material (Supplementary Fig. 1). Both orchards contain 10-year-old trees, planted at 3.5 × 1.5 spacing between rows and along the row, grafted onto M9 rootstock. Samples were collected over a development series: 40, 60, 80, 100, 120, 140, and 160 days after full bloom (DAFB). The sampling of the fruits was performed based on apple ripening parameters, such as days after full blossom, fresh weight, fruit diameter, starch content, fruit firmness, soluble solid contents, and dry matter. Sixty fruits were collected at each timepoint from three tree-rows (120 apple trees per row), and subsequently categorized at three biological replications (20 fruits per replication) at both regions. After separating the peel from the cortex (1 mm thick), the peel samples were frozen in liquid nitrogen and stored at −80 °C in order to be used for further analyses.

### Apple fruit ripening assessment

Fruit firmness was measured by the penetration of two opposite sides of each fruit, after removing 1 mm of peel tissue, using a texture analyzer (model 53205, T.R. Turoni srl, Forlì, Italy) with a 11 mm probe. Soluble solids content (SSC) was assessed in the apple juice using a refractometer (Atago PR-1, Atago Co Ltd., Tokyo, Japan), and fresh weight was calculated using a precision balance (Kern 440–47 N, KERN, Germany) as previously described^16^. Statistical analyses were performed by using SPSS 20.0 (SPSS, Chicago, IL, USA). Data were subjected to analysis of variance and least significant differences (LSD) at 5% level were used for means comparison (means consisted of the three biological replications).

### Starch determination

For starch analysis concentration, flesh tissue were processed as previously outlined^39^ with slight modifications. At first 0.5 g of freeze grinded tissue was placed in 15 mL plastic tube and 5 mL of 80% ethanol was added. Samples were incubated at 100 °C for 3 min and centrifuged at 10,000 × *g*, at room temperature for 5 min. Ethanol was allowed to evaporate under the hood, 5 mL of dH_2_O were added in the pellet, and subsequently the sample was incubated in a water bath set at 100 °C for 10 min. After cooling 1 mL of diluted sample were transferred to a glass vial and 10 μL of potassium iodide (0.01 M) were added. The sample was vortexed thoroughly and the absorbance was immediately measured at 590 nm using a Tecan Infinite M200 PRO microplate reader (Tecan, Männedorf, Switzerland). The final concentration was calculated as mg starch g^−1^ of fresh weight.

### Total anthocyanin concentration

Apples’ outer pericarp (peel) anthocyanins were extracted with 80% ethanol + 1% HCl, as previously described^40^. The total content of anthocyanins was determined by the pH differential assay and the results were expressed as μg g^−1^ fresh weight (FW). The final anthocyanin concentration was calculated as cyanidin-3*-O-*glucoside equivalent.

### Color parameters

External color was measured with a Minolta CR200 colorimeter (Minolta, Osaka, Japan) and the CIE (Commission International de l’Eclairage) parameters L* (lightness), a* (redness), and b* (yellowness), and hue angle (*h*^*o*^) were determined by performing measurements at two surfaces of each fruit and by calculating the mean between the measurements^41^.

### Analysis of primary metabolites in fruit development series by gas chromatography time-of-flight mass spectrometry

Primary metabolites of apple skin at 100, 120, 140, and 160 DAFB, were extracted and derivatized from 250 mg of freeze dried peel tissue, and thereafter analyzed using a gas chromatography time-of-flight mass spectrometry (GC–ToF–MS) as previously described^42^. Qualitative determination of peaks were performed using Tagfinder^43^ and their identity confirmed using the reference mass spectral in the MPI-MP Golm database^44^. All of the identified metabolite relative abundances were normalized in relation to the area of peak of the internal standard (ribitol) according to the procedure outlined in a previous study^45^. Values of 46 primary metabolites were obtained, these were also analyzed by one-way ANOVA followed by Duncan’s test to detect significant differences with *P* < 0.05. Details about the protocols^41^ of the metabolic profiling and the metabolic data are reported in Supplementary Table 1. Mean values of five independent samples for each developmental stage were expressed as the log_2_ transformed ratio between low altitude and to high altitude for each developmental stage, respectively.

### Ultra-performance liquid chromatography–tandem mass spectrometer analysis (UPLC–MS/MS) of polyphenolic compounds

The qualitative and quantitative polyphenolic profile was determined according to refs. ^41,46,47^. The fresh apple peel tissue was freeze dried and 100 mg of each sample were extracted with 4 mL methanol/water (80%). The obtained solutions were sonicated for 20 min, shaken for 3 h at 20 °C and left at 4 °C overnight in the dark. The methanolic extracts were filtered through a 0.22 µm polytetrafluoroethylene (PFTE) membrane filters into 1.5 ml glass vials and injected directly for analysis. Targeted UPLC–MS/MS analysis was performed on a Waters Acquity system (Milford, MA, USA) equipped with a binary pump, an autosampler, an online vacuum degasser, and a column compartment. The phenolic compounds were separated with the use of a Waters Acquity HSS T3 column 1.8 μm, 100 × 2.1 mm, set at 40 °C. The phenolic analysis was accomplished according to refs. ^41,46,47^. The determination of the anthocyanin compounds was performed in the same instrument using the method previously reported by refs. ^41,46,47^. The separation was carried out on a Waters RP Acquity UPLC^®^ BEH C18 column (130 Å, 2.1 × 150 mm, 1.7 µm), protected with a Waters Acquity UPLC^®^ BEH C18 precolumn (130 Å, 2.1 × 5 mm, 1.7 µm). A Waters Xevo TQMS instrument equipped with an electrospray (ESI) source was used for mass spectrometry detection. The processing of the data was carried out with Mass Lynx Target Lynx Application Manager (Waters) software. Three biological replicas were used for each developmental stage, and altitude region. The concentrations of Supplementary Table 2 are expressed as mg 100 g^−1^ FW.

### Comparative proteomic analysis of apple peel tissues

Apple peel tissue were isolated from apple fruit grown at low- and high-altitude regions at 140 and 160 DAFB developmental stages. The freeze dried peels were processed as outlined before^48^. The resulting protein extracts of three biological replicas of each condition were processed for proteomic analysis using the Filter Aided Sample Prep (FASP) mediated tryptic digestion protocol according to ref. ^16^. The obtaining peptide extracts were quantified using a nanodrop instrument and analyzed in two technical replicas by nano-LC coupled to HDAM Orbitrap mass spectrometer as described^16^. Finally, the acquired raw files were analyzed using the MaxQuant platform by searching against the downloaded *Malus Domestica* database from Genome Database of Rosaceae (GDR). The statistical evaluation of the different comparisons (high versus low altitude induced changes of both developmental stages) of the LFQ data was performed using the Perseus software. The identified proteins were annotated using available data of the GDR database by its KEGG orthologs and interPro domains. The proteomic dataset is available in PRIDE with identifier the PXD017037.

### Global metabolite-protein network analysis

The STITCH tool (version 5.0) was used^49,50^ to obtain additional correlations between metabolic and protein data for the subsequent functional validation of the metabolites and proteins that altered between the different environments (Supplementary Fig. 3).

## Supplementary information

Supplemental Data

Supplemental Table S1

Supplemental Table S2

Supplemental Table S3

Supplemental Table S4

## Data Availability

The proteomics data have been deposited via the PRIDE^51^ partner repository to the ProteomeXchange Consortium with dataset identifier PXD017037.
