# Supplementary material for: Decoding altitude-activated regulatory mechanisms occurring during apple peel ripening
Source: Hortic Res. 2020 Aug 1;7:120. doi: 10.1038/s41438-020-00340-x (PMC7395160; doi:10.1038/s41438-020-00340-x)
Supplement: Supplementary file 1 — Supplemental Data [file 41438_2020_340_MOESM1_ESM.docx]

# **Decoding altitude – activated regulatory mechanisms underlining apple peel coloration and ripening**

**Evangelos Karagiannis, Michail Michailidis, Georgia Tanou, Federico Scossa, Eirini Sarrou, George Stamatakis, Martina Samiotaki, Stefan Martens, Alisdair R. Fernie, Athanassios Molassiotis***

**Supplementary data**

Supplementary Figures 1 – 3 and Supplementary Files 1 – 4

**Supplementary Figure 1**

**Supplementary Figure 2**

**Supplementary Figure 3**

**Supplementary Figures**

**Supplementary Figure 1.** Temperature max- and min- at both altitude across apple various developmental stages.

**Supplementary Figure 2.** Subcellular localization of the identified proteins.

**Supplementary Figure 3.** Global network analysis of significantly affected metabolites and proteins by STITCH tool.

**Supplementary Files**

**Supplementary Table S1.** Apple peel primary metabolites affected by different altitude.

**Supplementary Table S2.** Apple peel secondary metabolites affected by different altitude.

**Supplementary Table S3.** Apple peel significantly deregulated proteins identified by label-free quantitative proteomics.

**Supplementary Table S4.** Proteins commonly altered at 140 and 160 DAFB between low- and high- altitude.
